# Association between the use of allopurinol and risk of increased thyroid-stimulating hormone level

**DOI:** 10.1038/s41598-021-98954-1

**Published:** 2021-10-13

**Authors:** Wona Choi, Yoon-Sik Yang, Dong-Jin Chang, Yeon Woong Chung, HyungMin Kim, Soo Jeong Ko, Sooyoung Yoo, Ji Seon Oh, Dong Yoon Kang, Hyeon-Jong Yang, In Young Choi

**Affiliations:** 1 Department of Medical Informatics, College of Medicine, The Catholic University of Korea, Seoul, Republic of Korea; 2 Department of Biomedicine and Health Sciences, The Catholic University of Korea, Seoul, Republic of Korea; 3 Department of Ophthalmology, Yeouido St. Mary’s Hospital, The Catholic University of Korea, Seoul, Republic of Korea; 4 Department of Ophthalmology and Visual Science, St. Vincent’s Hospital, College of Medicine, The Catholic University of Korea, Seoul, Republic of Korea; 5 Healthcare ICT Research Centre, Office of eHealth Research and Businesses, Seoul National University Bundang Hospital, Seongnam, Republic of Korea; 6 Department of Information Medicine, Big Data Research Centre, Asan Medical Centre, Seoul, Republic of Korea; 7 Drug Safety Monitoring Centre, Seoul National University Hospital, Seoul, Republic of Korea; 8 Department of Pediatrics, Soonchunhyang University College of Medicine, Asan, Republic of Korea

**Keywords:** Endocrinology, Health care, Medical research, Rheumatology

## Abstract

Allopurinol is the first-line agent for patients with gout, including those with moderate‐to‐severe chronic kidney disease. However, increased thyroid-stimulating hormone (TSH) levels are observed in patients with long-term allopurinol treatment. This large-scale, nested case–control, retrospective observational study analysed the association between allopurinol use and increased TSH levels. A common data model based on an electronic medical record database of 19,200,973 patients from seven hospitals between January 1997 and September 2020 was used. Individuals aged > 19 years in South Korea with at least one record of a blood TSH test were included. Data of 59,307 cases with TSH levels > 4.5 mIU/L and 236,508 controls matched for sex, age (± 5), and cohort registration date (± 30 days) were analysed. An association between the risk of increased TSH and allopurinol use in participants from five hospitals was observed. A meta-analysis (*I*^2^ = 0) showed that the OR was 1.51 (95% confidence interval: 1.32–1.72) in both the fixed and random effects models. The allopurinol intake group demonstrated that increased TSH did not significantly affect free thyroxine and thyroxine levels. After the index date, some diseases were likely to occur in patients with subclinical hypothyroidism and hypothyroidism. Allopurinol administration may induce subclinical hypothyroidism.

## Introduction

Gout is the most common form of inflammatory arthritis, caused by chronic elevation in serum uric acid levels above the saturation point of monosodium urate^1^. This leads to the deposition of monosodium urate crystals in the joints, tendons, and other tissues, triggering recurrent episodes of pronounced acute inflammation known as gout flares^2^.

In recent decades, gout incidence has increased in many countries. Although its prevalence in South Korea is lower than that in other countries, the incidence increased by 25% between 2009 and 2015, according to a study using the national health insurance claim database. Dietary factors, increasing age, and comorbid conditions were environmental influences on gout incidence^2,3^.

As the primary urate-lowering therapy, xanthine oxidase inhibitors (XOIs) such as allopurinol (the preferred first-line agent) or febuxostat^4^ are strongly recommended for all patients, including those with moderate‐to‐severe chronic kidney disease (CKD)^5^. According to previous studies, XOIs increase blood thyrotropin (TSH) levels^6–8^. Increased TSH levels (> 5.5 µIU/mL) were observed in patients on long-term treatment with allopurinol (5.8%) in a long-term, open-label extension study^9^. In a study by Perez-Ruiz et al.^7^, 88 patients receiving febuxostat and 87 patients receiving allopurinol were followed up for 12 months to measure changes in TSH levels. The authors found significant increases in TSH levels (> 5.5 µU/mL) in 3.4% of patients using allopurinol and 7.9% of patients using febuxostat. Increased TSH levels were not associated with changes in free thyroxine (FT4) levels^7^.

In 2017, the European Medicines Agency recommended that product information of allopurinol should be updated to include information regarding increased TSH levels in patients undergoing long-term treatment^10^. Information on increases in TSH appears on medication labels in Europe, including in the United Kingdom, France, Germany, Switzerland, and Italy^11–15^. In Korea, the label of allopurinol was updated to include this information in April 2020^16^. Despite the update on medication labels of allopurinol, there is no known evidence that allopurinol use is related to increased TSH levels in Korea.

Observational databases have different logical organisations and physical formats, and terminologies used to describe medicinal products and clinical conditions vary across sources^17^. A common data model (CDM) could be used to create a standardised data structure to utilise hospital data efficiently to overcome these difficulties. The CDM is a distributed database system of encrypted and de-identified information that identifies patients and converts their information to secondary data sources in a common format. Medical records are converted into standardised data in a common format and used as secondary data sources through the distributed research network.

This CDM allows for a range of standard queries and analytic methods developed centrally to be run seamlessly in both the distributed and centralised environments, potentially leading to rapid quality-assured results^18^. The MOA CDM from the Korea Institute of Drug Safety and Risk Management includes the Sentinel CDM from the US Food and Drug Administration and the Observational Medical Outcomes Partnership (OMOP) CDM from Observational Health Data Sciences and Informatics (OHDSI)^19^. The OMOP CDM is the most widely used model, developed and managed by the OHDSI in Korea.

In this retrospective, observational study targeting a large cohort of Korean population groups in multiple centres, we performed an analysis of the association between allopurinol and increased TSH levels using the OMOP CDM.

## Methods

### Data source and study design

This was a nested case–control study using distributed OMOP CDM databases loaded with data records from 19,200,973 patients based on electronic medical records (EMR) from seven hospitals. These data included demographic information, medical diagnoses, prescriptions, referrals, laboratory test results, and clinical values.

The Research Ethics Committee of the seven university hospitals (Seoul St. Mary’s Hospital, Seoul Asan Hospital, St. Vincent’s Hospital, Yeouido St. Mary’s Hospital, Soonchunhyang University Hospital Seoul, Seoul National University Bundang Hospital, and Seoul National University Hospital) approved the study. This study used CDM data, which were de-identified, and involved no more than minimal risk to subjects. The requirement for written informed consent was waived by the Research Ethics Committee of the Catholic Medical Centre and this study was in accordance with relevant guidelines and regulations.

### Selection of cases and controls

**Figure 1:**
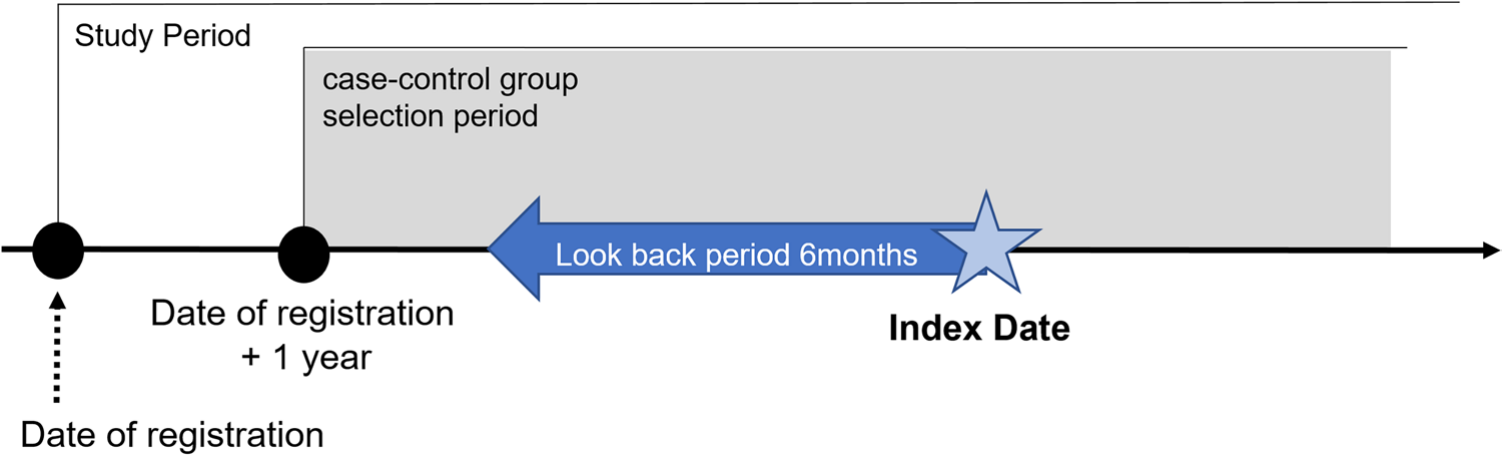
Study design.

This cohort included patients aged 19 years or older with at least one record of blood TSH test registered between 1 January 1997 and 18 September 2020. The date of registration was that of the first TSH test. Those who met any exclusion criteria during the entire period from the start date of the observation period to 1 year after the registration date were excluded. We excluded patients with TSH levels < 0.5 mIU/L or > 4.5 mIU/L; those diagnosed with thyroid diseases, Graves’ disease, pituitary tumour, pituitary gland dysfunction resulting in Sheehan’s syndrome, and those who underwent thyroidectomy. Patients receiving anti-thyroid medications, levothyroxine, thyroid supplements, or radioiodine therapy were also excluded (Supplementary Table S1). Case and control patients were selected 1 year after the date of registration (Fig. 1).

To reduce bias, we collected and reviewed the source values and standard codes of each hospital in advance and selected 5 out of 108 items for TSH. We confirmed that all seven hospitals participating in the study received laboratory accreditation from the Laboratory Medicine Foundation ensure the reliability of test result. Besides, we collected the normal range for TSH from the seven hospitals participating in the study. Then, we combined and compared the values in previous studies, and decided the cut off value TSH as 0.5 to 4.5 mIU/L with the help of an endocrinology specialist.

Patients whose TSH levels were > 4.5 mIU/L after 1 year of cohort registration were allocated to the case group, and those having levels > 4.5 mIU/L at the date of the first test were assigned to the index date of the case group. The patients in the control group who had TSH levels from 0.5 to 4.5 mIU/L were matched four times with the case group for sex, age (± 5), and date of cohort registration (± 30 days) using incidence density sampling without replacement. The control index date was assigned as the case group index date of the patients matching.

### Exposure

Details of medication prescriptions, including allopurinol were obtained (Supplementary Table S2). We selected the look-back period of allopurinol use for 6 months before the case–control index date and determined whether allopurinol was prescribed.

### Confounding variables

Confounding variables were selected by reviewing previous studies and expert consultations based on data available on the CDM network. We considered diseases and medications that could affect TSH levels as confounding variables and analysed the records for 6 months before selecting the case and control groups.

Diseases included 17 disease groups (myocardial infarction, congestive heart failure, peripheral vascular disease, cerebrovascular disease, hemiplegia or paraplegia, dementia, chronic pulmonary disease, rheumatologic disease, peptic ulcer disease, diabetes without chronic complications, diabetes with chronic complications, renal disease, any malignancy including leukaemia and lymphoma, metastatic solid tumour, mild liver disease, moderate or severe liver disease, and acquired immune deficiency syndrome/human immunodeficiency virus (AIDS/HIV) of the Charlson Comorbidity Index (CCI)^20–23^ and pituitary disease^24,25^. Medications included those that could affect TSH levels^26–34^ and medication used by gout patients, such as non-steroidal anti-inflammatories, acetaminophen, oxycodone, colchicine, and corticosteroids^35,36^ (Supplementary Table S3).

### Statistical analyses

Each hospital’s database was converted to the OMOP CDM version 5 format using the same table structures and standardised data. The association between allopurinol use and increment in TSH levels was analysed using a standardised analytical code. We used conditional logistic regression^37^ to estimate the adjusted odds ratio (OR) that increased TSH levels because of allopurinol exposure for the confounding variables. The overall effects were evaluated using a meta-analysis method, a statistical analysis combining the results of each hospital and a forest plot. Statistical approaches (*I*^2^ value test) were used to test for heterogeneity. We performed the following additional analyses for the case group that was prescribed allopurinol: (1) changes in TSH levels; (2) changes in FT4 and thyroxine (T4) levels; and (3) diseases diagnosed after the index date of the case group (the first increase in TSH). All analyses were performed using R 4.0.2 (http://www.R-project.org), and two-sided p-values lower than 0.05 were considered statistically significant.

## Results

### Clinical characteristics

The total population of all CDM databases was 19,200,973 patients from seven hospitals. A total of 2,298,492 patients were registered in the cohort according to the inclusion criteria, and 605,420 patients were excluded. Finally, the cohort comprised 1,693,072 patients.

**Figure 2:**
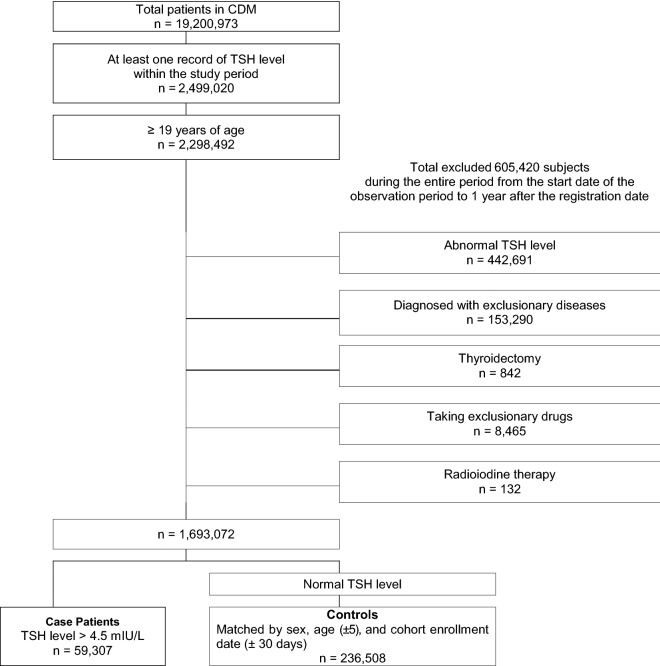
Flowchart of study participants in the study network. *CDM* Common data model, *TSH* Thyroid-stimulating hormone.

The case group had 59,307 case patients (TSH > 4.5 mIU/L) who were matched with 236,508 control patients for sex, age (± 5), and the date of cohort registration (± 30 days) using incidence density sampling without replacement after 1 year from the date of cohort registration. Detailed information regarding the number of cases and controls is described in Fig. 2.

Table 1 presents the clinical characteristics of the study population. All medications that were considered confounding variables showed significant differences between the case and control groups. Therefore, cases were found to more likely use the medication than controls. Among the diseases considered confounding variables, all diseases showed significant differences between the case and control groups. The case group had a higher incidence of disease than the control group.

**Table 1:** Characteristics of the study population.

	Total		Case patients		Controls		χ^2^
	n	(%)	n	(%)	n	(%)	*p* value
Total	295,815	(100)	59,307	(20.05)	236,508	(79.95)	0.510
**Sex**							
Male	114,732	(38.79)	23,039	(38.85)	91,693	(38.77)	0.730
Female	181,083	(61.21)	36,268	(61.15)	144,815	(61.23)	
**Age (years)**							
Mean (SD)	55.23	(13.06)	55.63	(13.26)	55.13	(13.00)	
19–29	7435	(2.51)	1500	(2.53)	5935	(2.51)	< 0.001
30–39	26,426	(8.93)	5031	(8.48)	21,395	(9.05)	
40–49	65,489	(22.14)	12,825	(21.62)	52,664	(22.27)	
50–59	88,710	(29.99)	17,934	(30.24)	70,776	(29.93)	
60–69	63,352	(21.42)	12,537	(21.14)	50,815	(21.49)	
70–79	34,569	(11.69)	7119	(12.00)	27,450	(11.61)	
≥ 80	9834	(3.32)	2361	(3.98)	7473	(3.16)	
**Medication**							
Tyrosine kinase inhibitors	382	(0.13)	227	(0.38)	155	(0.07)	< 0.001
Cancer immunotherapy	70	(0.02)	62	(0.10)	8	(0.00)	< 0.001
Anti-tuberculosis medications	462	(0.16)	204	(0.34)	258	(0.11)	< 0.001
Dobutamine	287	(0.10)	197	(0.33)	90	(0.04)	< 0.001
Octreotide	42	(0.01)	18	(0.03)	24	(0.01)	< 0.001
Interferon-α	133	(0.04)	111	(0.19)	22	(0.01)	< 0.001
Amiodarone	1613	(0.55)	1282	(2.16)	331	(0.14)	< 0.001
Azathioprine	380	(0.13)	135	(0.23)	245	(0.10)	< 0.001
Mercaptopurine	17	(0.01)	7	(0.01)	10	(0.00)	0.029
Warfarin	3015	(1.02)	1313	(2.21)	1702	(0.72)	< 0.001
Dopamine	444	(0.15)	271	(0.46)	173	(0.07)	< 0.001
Metformin	11,099	(3.75)	4016	(6.77)	7083	(2.99)	< 0.001
NSAIDs	28,054	(9.48)	10,867	(18.32)	17,187	(7.27)	< 0.001
Acetaminophen	10,961	(3.71)	5235	(8.83)	5726	(2.42)	< 0.001
Oxycodone	1346	(0.46)	642	(1.08)	704	(0.30)	< 0.001
Colchicine	438	(0.15)	166	(0.28)	272	(0.12)	< 0.001
Corticosteroid	10,474	(3.54)	4437	(7.48)	6037	(2.55)	< 0.001
**Disease**							
Panhypopituitarism	42	(0.01)	25	(0.04)	17	(0.01)	< 0.001
Myocardial Infarction	1362	(0.46)	480	(0.81)	882	(0.37)	< 0.001
Congestive heart failure	2390	(0.81)	1221	(2.06)	1169	(0.49)	< 0.001
Peripheral vascular disease	1497	(0.51)	561	(0.95)	936	(0.40)	< 0.001
Cerebrovascular disease	5514	(1.86)	1793	(3.02)	3721	(1.57)	< 0.001
Dementia	2440	(0.82)	632	(1.07)	1808	(0.76)	< 0.001
Chronic pulmonary disease	3021	(1.02)	1180	(1.99)	1841	(0.78)	< 0.001
Rheumatologic disease	1710	(0.58)	597	(1.01)	1113	(0.47)	< 0.001
Peptic ulcer disease	3134	(1.06)	1140	(1.92)	1994	(0.84)	< 0.001
Mild liver disease	2840	(0.96)	1115	(1.88)	1725	(0.73)	< 0.001
Diabetes without chronic complications	12,413	(4.20)	4905	(8.27)	7508	(3.17)	< 0.001
Diabetes with chronic complications	7832	(2.65)	3199	(5.39)	4633	(1.96)	< 0.001
Hemiplegia or paraplegia	292	(0.10)	147	(0.25)	145	(0.06)	< 0.001
Renal disease	3637	(1.23)	1834	(3.09)	1803	(0.76)	< 0.001
Any malignancy	13,631	(4.61)	6912	(11.65)	6719	(2.84)	< 0.001
Moderate or severe liver disease	225	(0.08)	124	(0.21)	101	(0.04)	< 0.001
Metastatic solid tumour	1726	(0.58)	1259	(2.12)	467	(0.20)	< 0.001
AIDS/HIV	67	(0.02)	25	(0.04)	42	(0.02)	< 0.001
**CCI group**							
0	264,455	(89.4)	45,957	(77.49)	218,498	(92.39)	< 0.001
1	8634	(2.92)	3010	(5.08)	5624	(2.38)	
2	15,722	(5.31)	6658	(11.23)	9064	(3.83)	
> 3	7004	(2.37)	3682	(6.21)	3322	(1.40)	

*SD* Standard deviation, *NSAIDs* Nonsteroidal anti-inflammatory drugs, *AIDS/HIV* Acquired immune deficiency syndrome/human immunodeficiency virus, *CCI* Charlson comorbidity index.

### Association between allopurinol use and increased TSH

**Figure 3:**
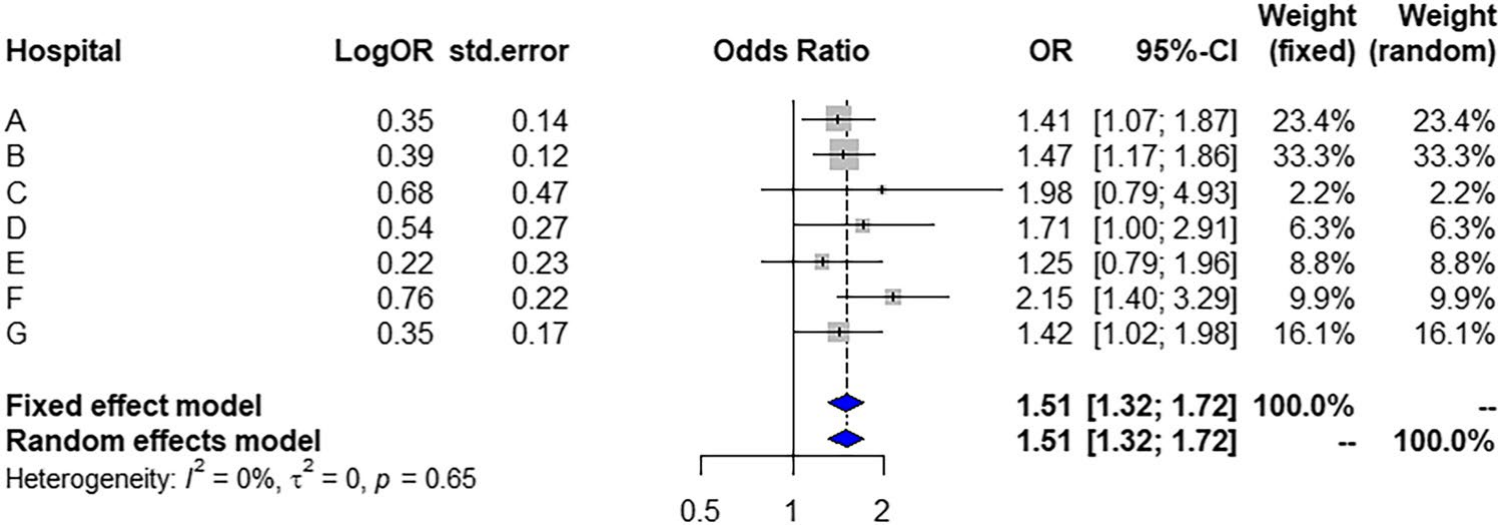
Forest plot. *OR* Odds ratio, *CI* Confidence interval.

Allopurinol use was associated with an increased risk of elevated TSH in five of seven hospitals (Table 2). Figure 1 presents the adjusted OR of allopurinol use for increased TSH levels from the meta-analysis. The OR was 1.51 (95% confidence interval, 1.32–1.72) in both the fixed effects and random effects models. In the meta-analysis, the fraction of variance due to heterogeneity was estimated using the statistic *I*^2^; the *I*^2^ value was zero (Fig. 3).

**Table 2:** Crude and adjusted odds ratios (ORs) showing the association between allopurinol use and increased thyroid-stimulating hormone (TSH) levels.

Hospital	Allopurinol use	Case patients		Controls		Crude OR		Adjusted OR	
		n = 59,307		n = 236,508					
		n	%	n	%	OR	95% CI	OR	95% CI
A		6646		26,569					
	No	6520	98.10	26,419	99.44				
	Yes	126	1.90	150	0.56	3.40	(2.68, 4.32)	1.41	(1.07, 1.87)
B		29,868		119,047					
	No	29,694	99.42	118,844	99.83				
	Yes	174	0.58	203	0.17	3.43	(2.80, 4.20)	1.47	(1.17, 1.86)
C		2360		9422					
	No	2347	99.45	9409	99.86				
	Yes	13	0.55	13	0.14	4.01	(1.86, 8.66)	1.98	(0.79, 4.93)
D		1094		4354					
	No	1049	95.89	4314	99.08				
	Yes	45	4.11	40	0.92	4.63	(3.01, 7.12)	1.71	(1.00, 2.91)
E		3805		15,082					
	No	3737	98.21	15,030	99.66				
	Yes	68	1.79	52	0.34	5.26	(3.66, 7.56)	1.25	(0.79, 1.96)
F		6882		27,457					
	No	6822	99.13	27,404	99.81				
	Yes	60	0.87	53	0.19	4.55	(3.14, 6.59)	2.15	(1.40, 3.29)
G		8652		34,577					
	No	8570	99.05	34,458	99.66				
	Yes	82	0.95	119	0.34	2.77	(2.09, 3.67)	1.42	(1.02, 1.98)

*CI* Confidence interval.

### Additional analysis

We performed an additional analysis for patients who used allopurinol. The cohort end date was set as the date 1 year after the last use of allopurinol and the changes in TSH levels, TSH value at the date of cohort registration, index date of the case, maximum TSH value after the case index date, and mean TSH value after the case index date were analysed.

The mean TSH level was 2.49 mIU/L at the date of cohort registration and 10.54 mIU/L at the index date in the case group. The mean maximum TSH level was 12.52 mIU/L. The mean TSH level between the index date and cohort end date was 7.64 mIU/L in the case group (Table 3).

**Table 3:** Changes in thyroid-stimulating hormone (TSH) levels.

Hospital	Variable	Cohort registration	Index date	Max	Mean
Total	Mean ± SD	2.49 ± 1.06	10.54 ± 18.11	12.52 ± 19.72	7.64 ± 15.15
	n	568	568	568	2021
A	Mean ± SD	2.48 ± 1.02	8.1 ± 10.18	9.12 ± 10.7	5.94 ± 7.93
	n	126	126	126	413
B	Mean ± SD	2.31 ± 1.04	11.91 ± 18.5	15.5 ± 22.18	9.59 ± 17.51
	n	174	174	174	642
C	Mean ± SD	2.28 ± 0.87	6.18 ± 1.93	6.2 ± 1.92	5.53 ± 2.07
	n	13	13	13	20
D	Mean ± SD	2.44 ± 0.93	11.65 ± 22.63	13.25 ± 22.91	8.25 ± 15.61
	n	45	45	45	170
E	Mean ± SD	2.47 ± 1.07	8.87 ± 12.5	10 ± 12.77	5.73 ± 8.28
	n	68	68	68	199
F	Mean ± SD	2.79 ± 1.46	7.99 ± 12.33	8.63 ± 13.79	5.67 ± 9.69
	n	60	60	60	250
G	Mean ± SD	2.78 ± 1.02	14.69 ± 39.86	16.92 ± 41.06	8.42 ± 28.85
	n	82	82	82	327

*SD* Standard deviation.

We analysed the FT4 and T4 values before and after the index date of the case group. The mean FT4 level was within the normal reference range after the case group’s index date in all hospitals. The mean T4 level was within the normal reference range in all hospitals (Table 4).

**Table 4:** Changes in free thyroxine (FT4) and thyroxine (T4) levels.

Hospital	Variable	FT4 (ng/dL)		T4 (μg/dL)	
		Before	After	Before	After
		Index date	Index date	Index date	Index date
Total	Mean ± SD	1.23 ± 0.32	1.2 ± 0.2	8.03 ± 1.25	7.27 ± 1.23
	N	513	526	62	27
A	Mean ± SD	1.22 ± 0.21	1.17 ± 0.24	8.75 ± 2.41	6.82 ± 1.98
	n	112	117	19	9
B	Mean ± SD	1.31 ± 0.57	1.3 ± 0.26	8.12 ± 1.87	6.66 ± 2.49
	n	163	167	29	13
C	Mean ± SD	1.1 ± 0.14	0.9 ± 0.18	8.38 ± 1.76	10.53
	n	13	13	2	1
D	Mean ± SD	1.15 ± 0.29	1.11 ± 0.28		
	n	44	45		
E	Mean ± SD	1.23 ± 0.41	1.2 ± 0.27	5.96 ± 2.16	6.21 ± 0.46
	n	65	59	6	3
F	Mean ± SD	1.29 ± 0.3	1.24 ± 0.18	7.33 ± 2.51	4.5
	n	39	44	6	1
G	Mean ± SD	1.11 ± 0.3	1.11 ± 0.27		
	n	77	81		
Reference range	FT4: 0.9–1.7 ng/dL			T4: 5.0–11.0 μg/dL	

*SD* Standard deviation, *Ft4* Free thyroxine, *T4* Thyroxine.

We analysed new diseases that occurred after the case group’s index date based on the International Classification of Diseases 10th Revision (ICD-10) code in the case of patients who used allopurinol. CKD (N18) was the most common (in 133 cases), followed by gastro-oesophageal reflux disease (GERD) (K21) in 117, chronic ischaemic heart disease (IHD) (I25) in 113, type 2 diabetes mellitus (T2DM) (E11) in 97, and gastritis and duodenitis (K29) in 83 (Table 5).

**Table 5:** Diseases diagnosed after increased thyroid-stimulating hormone (TSH) levels.

ICD-10 code	Disease	Total
N18	Chronic kidney disease	133
K21	Gastro-oesophageal reflux disease	117
I25	Chronic ischaemic heart disease	113
E11	Type 2 diabetes mellitus	97
K29	Gastritis and duodenitis	83
E13	Other specified diabetes mellitus	71
I48	Atrial fibrillation and flutter	63
K59	Other functional intestinal disorders	62
M1A	Chronic gout	60
J30	Vasomotor and allergic rhinitis	59
E03	Other hypothyroidism	57
G47	Sleep disorders	52
H35	Other retinal disorders	50
I50	Heart failure	50
H04	Disorders of lacrimal system	49
E87	Other disorders of fluid, electrolyte, and acid–base balance	44
M54	Dorsalgia	44
I10	Essential (primary) hypertension	43
E78	Disorders of lipoprotein metabolism and other lipidaemias	40
J06	Acute upper respiratory infections of multiple and unspecified sites	39
Omitted 578 items below		3462

*ICD-10* International classification of diseases 10th revision.

## Discussion

We found an association between allopurinol use and an increased TSH level using a CDM based on the EMRs of 19,200,973 patients in the distributed databases of seven hospitals. All medications and diseases considered confounding variables showed significant differences between the case and control groups. The adjusted OR for the risk between increased TSH and allopurinol use in each hospital, after an aggregated meta-analysis, was 1.51 (95% CI; 1.32–1.72). Regarding the additional analysis of patients who used allopurinol, the mean maximum TSH level was 12.52 mIU/L and the highest level was 16.92 mIU/L at hospital G. Since free T4 levels were all within the normal reference range even after the case group index date (that is, when the TSH level increased), the possibility of subclinical hypothyroidism (SCH) could be suspected^38,39^.

Serum TSH measurement is the single most reliable test to diagnose all common forms of hypothyroidism and hyperthyroidism^40^. The best way to initially test thyroid function is to measure TSH levels in a blood sample^41^. Most laboratories report a normal TSH reference range of 0.4–0.5 mIU/L on the lower end and 4–5.5 mIU/L on the upper end of the range^42,43^. Primary hypothyroidism is manifested by elevated serum TSH with low serum FT4 levels. In SCH, although FT4 levels were within the normal reference range, there were elevations in TSH^44^.

Our findings are consistent with those of Perez-Ruiz et al.^7^ and Faisal et al.^8^. These studies showed that allopurinol use affected TSH levels and was associated with the onset of SCH. We identified diseases that occurred after the case group’s index date based on the ICD-10 codes for patients who used allopurinol. They were CKD, GERD, chronic IHD, T2DM, gastritis, and duodenitis, among others. These diseases have a high prevalence in patients with SCH or hypothyroidism, as reported by previous studies. These findings accorded with those of previous studies of associations between SCH or hypothyroidism and CKD^45–48^, GERD^49,50^, IHD^51–54^ and T2DM^55,56^.

Other studies have suggested that SCH is associated with an increased risk of coronary heart disease (CHD) events. In particular, CHD mortality rates increased in those with higher TSH levels, particularly in those with TSH levels of 10 mIU/L or greater^52,57,58^. Treatment might be needed for patients with SCH and serum TSH levels of 10 mIU/L^38^.

Our study has several limitations. This observational study showed associations, but it was unable to determine causality. Traditional medical record reviews provide detailed clinical information, whereas the database of the CDM deidentifies personal information to protect patient privacy. Additional information was not available in this study^59^. Due to the lack of laboratory results, we could not consider the thyroid autoimmune reaction and iodine status as covariates in this study. It is important to consider these in the study design^60,61^. Laboratory tests for a thyroid autoimmune reaction detect the presence and measure the number of specific thyroid autoantibodies in the blood. Therefore, if laboratory values are out of normal range in a patient who uses the allopurinol, we can suspect that allopurinol causes autoimmune diseases. Although the analyses accounted for a wide range of potential confounding variables, there is potential for residual confounding and indication biases in observational studies. Also a retrospective study, it is important to standardize and confirm the laboratory items using a laboratory study because the study is related to the reliability of the inspection items and results. Therefore, a further study that reflects these considerations is required.

To date, the understanding of pathogenetic effects and mechanisms of allopurinol on TSH limited. An experimental study on rats revealed decreases of both triiodothyronine and thyroxine levels in thyrotoxicosis rats receiving allopurinol compared with untreated thyrotoxicosis rats, and the authors suggested an association between allopurinol and the biosynthesis of thyroid hormones^6^. However, this theory has been not proven in clinical studies. Only a few reports suggested possible mechanisms in human studies. Faisal et al. guessed that allopurinol use might affect the enlarged thyroid gland, change echogenicity, and increase blood supply with nodule formation^8^. On the other hand, Perez-Ruiz et al. assumed that changes in TSH might be directly associated with inhibition of xanthine oxidase because a higher dose had a greater impact on TSH in their study^7^.

Nevertheless, we provided evidence for the safety of the medication using multicentre CDM data. Though allopurinol labels have recently changed in Korea based on previous research and approval from other countries, there has been a lack of relevant large-scale analysis to date.

SCH implies an absence of symptoms; however, it is perhaps better thought of as mild hypothyroidism^62^. Moreover, even mild hypothyroidism can progress to overt hypothyroidism^63,64^. Hypothyroidism is permanent in most patients and therefore requires hormone replacement over a lifetime^65^. Therefore, it is essential to provide evidence for the safety of the medication.

The risk of an increased TSH level was significantly higher in the allopurinol use group than in the allopurinol non-use group based on a CDM built using a large-scale hospital EMR, which is highly efficient. Additional analysis confirmed the potential for the development of SCH. TSH is a sensitive indicator, which detects changes in thyroid function. Therefore, we suggest that patients using allopurinol pay attention and observe changes in thyroid function. Thyroid function changes should be monitored because related diseases do not occur immediately. Continuous follow-up is necessary, even if no specific clinical symptoms appear immediately.

## Supplementary Information

Supplementary Information.

## Data Availability

The data that support the findings of this study are available from each hospital. Analyses were performed locally, and the patient-level data are not readily available to be shared. The analytic code is, however, available at: https://github.com/WonaChoi/Allopurinol-TSH.
