# Supplementary material for: Association between the use of allopurinol and risk of increased thyroid-stimulating hormone level
Source: Sci Rep. 2021 Oct 13;11:20305. doi: 10.1038/s41598-021-98954-1 (PMC8514499; doi:10.1038/s41598-021-98954-1)
Supplement: Supplementary file 1 — Supplementary Information. [file 41598_2021_98954_MOESM1_ESM.doc]

Supplementary Information

Supplemenatary to: Association between the use of allopurinol and risk of increased thyroid-stimulating hormone level

Authors : Wona Choi, Yoon-Sik Yang, Dong-Jin Chang, Yeon Woong Chung, HyungMin Kim, Soo Jeong Ko, Sooyoung Yoo, Ji Seon Oh, Dong Yoon Kang, Hyeon-Jong Yang, In Young Choi*

Supplementary Table S1. Concept identification codes for cohort inclusion/exclusion criteria

| **Domain** | **Name** | **Code** | **Vocabulary** |
| --- | --- | --- | --- |
| Measurement | Thyrotropin | 3023399, 3009201, 3027700, 3019170, 3019762 | LOINC |
| Condition | Diabetes insipidus | 30968 | SNOMED |
|  | Hypothyroidism | 140673 |  |
|  | Hypothalamic syndrome | 4008579 |  |
|  | Thyroid nodule | 4030041 |  |
|  | Neoplasm of the pituitary gland | 4130061 |  |
|  | Neoplasm of the thyroid gland | 4131909 |  |
|  | Iodine deficiency syndrome | 4129370 |  |
|  | Hypopituitarism | 4254542 |  |
| Procedure | Iodine 131 therapy | 4036252 | SNOMED |
| Drug | carbimazole | 19040606 | RxNorm |
|  | methimazole | 1504620 |  |
|  | propylthiouracil | 1554072 |  |
|  | levothyroxine | 1501700 |  |

Descendants were included.

Vocabulary was defined by the logical observation identifier names and codes (LOINC), systematized nomenclature of medicine (SNOMED), and RxNorm.

Supplementary Table S2. Concept identification codes for allopurinol

| **Domain** | **Name** | **Code** | **Vocabulary** |
| --- | --- | --- | --- |
| Drug | Allopurinol | 1167322 | RxNorm |

Descendants were included.

Vocabulary was defined by the RxNorm.

Supplementary Table S3. Concept identification codes for confounding variables

| **Medication** | | | |
| --- | --- | --- | --- |
| **Domain** | **Category** | **Code** | **Vocabulary** |
| Drug | Cancer immunotherapy | 40238188, 1312706, 45775965, 45892628, 45892531, 42629079 | RxNorm |
|  | Tyrosine kinase inhibitors | 43533090, 40238052, 42709322, 1325363, 1336539, 1359548, 1363387, 1358436, 1319193, 40242675, 1394023, 1304107 |  |
|  | Anti-tuberculosis medications | 19135812, 1710446, 19026710,  1749301, 1763204, 1759455, 19078424, 1782521, 43012518 |  |
|  | Dobutamine | 1337720 |  |
|  | Octreotide | 1522957 |  |
|  | Interferon-α | 40053021, 1385645, 1379969, 19044394, 1380068, 1714165, 1797155, 1781314 |  |
|  | Amiodarone | 1309944 |  |
|  | Azathioprine | 19014878 |  |
|  | Mercaptopurine | 1436650 |  |
|  | Warfarin | 1310149 |  |
|  | Dopamine | 1337860 |  |
|  | Metformin | 1503297 |  |
|  | NSAIDs | 1102917, 1136980, 1112807, 1126128, 1124300, 1156378, 1195492, 1236607, 1153928, 1137460, 1180182, 1178663, 1177480, 1146810, 1185922, 1115008, 1150345, 1113648, 1118045, 1197736 |  |
|  | Acetaminophen | 1125315 |  |
|  | Oxycodone | 1124957 |  |
|  | Colchicine | 1101554 |  |
|  | Corticosteroid | 1551099, 1518254, 920458, 903963, 975125, 1506270, 1507705, 19086888, 1550557 |  |
| **Disease** | | | |
| **Domain** | **Category** | **Code** | **Vocabulary** |
| Condition | Panhypopituitarism | 30365 | SNOMED |
|  | Myocardial Infarction | 4329847 |  |
|  | Congestive heart failure | 316139 |  |
|  | Peripheral vascular disease | 321052 |  |
|  | Cerebrovascular disease | 434056, 381591 |  |
|  | Dementia | 4182210 |  |
|  | Chronic pulmonary disease | 4063381 |  |
|  | Rheumatologic disease | 80800, 257628, 256197, 80809, 255348, 134442 |  |
|  | Peptic ulcer disease | 4247120 |  |
|  | Mild liver disease | 4064161, 4212540 |  |
|  | Diabetes without chronic complications | 201820 |  |
|  | Diabetes with chronic complications | 442793, 443767, 4192279 |  |
|  | Hemiplegia or paraplegia | 192606, 374022 |  |
|  | Renal disease | 4030518 |  |
|  | Any malignancy | 443392 |  |
|  | Moderate or severe liver disease | 24966, 192680, 4029488, 4245975 |  |
|  | Metastatic solid tumour | 432851 |  |
|  | AIDS/HIV | 439727, 30365 |  |

Descendants were included.

NSAIDs: nonsteroidal anti-inflammatory drugs; AIDS/HIV: acquired immune deficiency syndrome/human immunodeficiency virus

The vocabulary was defined by the Systematized Nomenclature of Medicine (SNOMED), and RxNorm.
